# Atypical Enteropathogenic *Escherichia coli*: Typical Pathogens?

**DOI:** 10.3201/eid1204.060125

**Published:** 2006-04

**Authors:** James P. Nataro

**Affiliations:** * University of Maryland School of Medicine, Baltimore, Maryland, USA

**Keywords:** Escherichia coli, EPEC, commentary

*Escherichia coli* is both the most abundant facultative commensal of the human gastrointestinal tract and the most common bacterial cause of human diarrhea (*1*). However, precise recognition of *E. coli* pathotypes remains problematic. Enteropathogenic *E. coli* (EPEC), classically associated with outbreaks of infant diarrhea, harbors distinctive chromosomal (the locus of enterocyte effacement, or LEE island) and plasmidborne (residing on the EPEC adherence factor, or EAF, plasmid) virulence factors, which are linked by common gene regulators (*1*). At the Second International Conference on EPEC, held in São Paulo, Brazil, in 1995, the foremost authorities in the field proclaimed the global importance of such "typical" EPEC (tEPEC) but pondered the clinical relevance of strains carrying only the LEE island (dubbed at that conference "atypical EPEC," or aEPEC) (*2*). Had aEPEC lost the EAF plasmid? Had it incidentally acquired only fragments or incomplete packages of virulence-associated genes? Or were some aEPEC true pathogens of humans or animals?

In this issue of Emerging Infectious Diseases, Nguyen et al. propose a distinct role for aEPEC in human infection (*3*). Previously, these investigators reported a high prevalence of aEPEC among pediatric diarrhea patients in Melbourne, including both infants and older children (in contrast to the strong tendency for infants to be infected with tEPEC) (*4*). Now these authors show that, in contrast to patients infected with other pathogens, patients infected with aEPEC are far more likely to experience diarrhea past 14 days, the point long recognized as a clinical watershed that heralds increased risk for illness and death. aEPEC's prevalence among diarrhea patients, the pathogen's strong association with diarrheal symptoms, and the infection's distinctively persistent nature argue for a high disease burden in Melbourne. Although the authors define aEPEC strictly on the basis of positivity for the LEE *eae* gene and failure to amplify a *bfpA* pilin gene (not assessing additional plasmid loci), the absence of tEPEC serotypes and the occurrence of disease in children older than infants suggest that these are indeed aEPEC.

This communication also illustrates 2 principles that should be recognized more generally with regard to diarrheogenic *E. coli*: 1) one can authoritatively implicate a particular strain as a pathogen by (only) outbreak implication or by volunteer studies, but one cannot definitively prove by any data that a putative pathotype is not a human pathogen; and 2) the implication of pathogenicity for 1 strain is not sufficient to implicate any similar strain as a pathogen. Thus, once definitive evidence is presented that a particular strain is pathogenic, the challenge is to determine the genotypic or phenotypic fingerprints that permit extrapolation to other strains. This obstacle has long proved daunting for the enteroaggregative pathotype (shown to be pathogenic in both volunteer studies and outbreaks), and it may prove similarly frustrating for aEPEC.

Diarrheal epidemiology studies today are able to identify a likely etiologic agent for most patients, in contrast to studies of 20 years ago, because of improved diagnostic tests and the identification of new pathogens, but the task is not finished. Additional studies are needed in many places to refine our understanding of putative and emerging pathogens and to determine their full epidemiologic roles.
